# Demographic and obstetric factors affecting women’s sexual functioning during pregnancy

**DOI:** 10.1186/s12978-015-0065-0

**Published:** 2015-08-19

**Authors:** Kobra Abouzari-Gazafroodi, Fatemeh Najafi, Ehsan Kazemnejad, Parvin Rahnama, Ali Montazeri

**Affiliations:** Department of Midwifery, School of Nursing and Midwifery, Guilan University of Medical Sciences, Rasht, Iran; Department of Biostatistics, School of Nursing and Midwifery, Guilan University of Medical Sciences, Rasht, Iran; Department of Midwifery, Faculty of Nursing and Midwifery, Shahed University, Tehran, Iran; Mental Health Research Group, Health Metrics Research Centre, Iranian Institute for Health Sciences Research, ACECR, Tehran, Iran; Faculty of Humanity Sciences, University of Science & Culture, ACECR, Tehran, Iran

## Abstract

**Background:**

Sexual desire and frequency of sexual relationships during pregnancy remains challenging. This study aimed to assess factors that affect women’s sexual functioning during pregnancy.

**Methods:**

This was a cross sectional study carried out at prenatal care clinics of public health services in Iran. An author-designed structured questionnaire including items on socio-demographic characteristics, obstetric history, the current pregnancy, and women’s sexual functioning during pregnancy was used to collect data. The generalized linear model was performed in order to find out factors that affect women’s sexual functioning during pregnancy.

**Results:**

In all, 518 pregnant women participated in the study. The mean age of participants was 26.4 years (SD = 4.7). Overall 309 women (59.7 %) scored less than mean on sexual functioning. The results obtained from generalized linear model demonstrated that that lower education, unwanted pregnancy, earlier stage of pregnancy, older age, and longer duration of marriage were the most important factors contributing to disturbed sexual functioning among couples.

**Conclusion:**

The findings suggest that sexual function during pregnancy might be disturbed due to several factors. Indeed issues on sexual relationship should be included as part of prenatal care and reproductive health programs for every woman.

## Background

Pregnancy is considered as one of the most critical periods in a woman’s life. During this period the couples’ sexual relationship and sexual function might be affected for several reasons [1–4]. Also it was reported that 68 % of young mothers did not receive any information about sexual relationships during pregnancy [5]. On the other hand it was determined that sexual intercourse at term is a stimulus for the induction of delivery that leads to decrease requirement for labor induction [6].

Pregnancy has been verified that plays role in the decreased sexual function [2, 7, 8]. Furthermore, it has been found that disrupted sexual functioning during pregnancy was related to Women’s tiredness, nausea and lack of sexual interest [2], high number of children [9–11], cultural beliefs, myths, and taboos about sexual behavior during pregnancy [12]. It is argued that the sexual perceptions is an important part of sexual function during pregnancy and sexuality might be influenced by desire from the partner, feelings of attractiveness, and fear of sexual intercourse [13, 14]. However different predictors of sexual functioning during pregnancy were reported. A study reported that the third trimester was the independent variable for both decreased sexual activity frequency and sexual function scores in pregnancy [15]. In addition a recent publication found that satisfaction with body image and body image self-consciousness were related to sexual satisfaction during pregnancy. Even, the study suggested that other aspects of partnership, such as communication, appeared to be much more important predictors of sexual satisfaction than body image variables. The best predictor of sexual frequency was fear that intercourse might harm the fetus [16]. Similarly a study reported that unsatisfying partner relationship was a significant factor affecting the quality of sexual life during pregnancy [17].

In general the sexual problems that are commonly reported by pregnant women include reduction in sexual desire, enjoyment, coital frequency and overall decline in sexual activities [7]. In addition the result of studies indicated that sexual dysfunction increases as pregnancy progresses [18, 19].

An Iranian descriptive study reported that women felt guilt, feared of pre-term labor and experienced post-coital stomachache, backache and vaginal irritation while they had sexual activity during pregnancy [20]. Sexual satisfaction and its related factors also were studied in another study. The findings indicated that there was a significant relationship between sexual satisfaction during pregnancy and several factors including women's age, their husbands' age, length of marriage, occupation, having pregnancy complication and concerns about fetus [21]. The aim of this study was to assess the most important factors affecting women’s sexual function during pregnancy in order to develop essential information for improving women’s sexual health.

## Methods

### Design and participants

This was a cross-sectional study carried out in Guilan, a province in north, Iran, from September 2010 to March 2011. A sample of women attending all five prenatal care clinics of public health services in Guilan were entered into the study. In all 543 pregnant women were approached. Criteria for inclusion were: being pregnant in any trimester of pregnancy, and being in the age group 18 to 35 years old. The gestational age was determined from the last menstrual cycle or was verified with ultrasound scan measurements. Women were excluded from the study if had complications including threatened abortion, hypertension in pregnancy, placenta previa, premature labor and medications that might have a negative effect on sexual function. Data were collected in a comfortable setting using a structured questionnaire at the prenatal care clinics.

### Questionnaire

The questionnaire consisted of two parts. The first part included questions about socio-demographic characteristics, obstetric history and medical details of the current pregnancy. The second section was a structured questionnaire including items on couples’ sexual functioning during pregnancy. An author-designed structured questionnaire derived from the literature [14, 22, 23]. It contained 17 questions measuring two concepts: Sexual satisfaction and Sexual worries. Sexual satisfaction included items on sexual desire (4 items), sexual excitement (3 items), and sexual enjoyment (3 items). Sexual worries included items on dyspareunia (3 item), and fetal injury concern (4 items). Each item was rated on a 5-point Likert scale ranging from 0 to 4, giving a total score ranging from 0 to 68. The higher scores indicated a better sexual function. The internal consistency of the questionnaire was assessed using the Cronbach's α coefficient and it was 0.84 indicating a satisfactory result [24]. For content validity, an expert committee consisting of two sexologists, five obstetricians and three midwives reviewed the questionnaire in order to assess if questions measured what they were intended to measure. Finally to ensure face validity, the questionnaire was given to 10 pregnant women and it was found that the items were clear enough to be understood without difficulty and that the questions were simple enough to be rated.

### Analysis

Descriptive statistics was used to explore the data. Then, the generalized linear model analysis (GLM) was performed in order to indicate factors predicting sexual dysfunction during pregnancy. We used generalized linear model for two reasons: first, the distribution of the dependent or response variable can be non-normal, and second, the dependent variable values are predicted from a linear combination of predictor variables, which are ‘connected’ to the dependent variable [25]. For this study women’s sexual functioning score was considered as outcome variable and women’s characteristics were considered as independent factors. The level of significance was set at 5 %. The B coefficient and 95 % confidence intervals was reported as indication of any association between dependent and independent variables. The SPSS version 16 was used to analyze the data.

### Ethics

Ethics committee of Guilan University of Medical Sciences approved the study. We obtained written informed consent from participants after comprehensive explanation of procedure involved.

## Results

### The study sample

In all, 518 agreed to participate in the study, giving a response rate of 95 % (518/543). The mean age of participants was 26.4 (SD = 4.7) years. All women were married and 62 % were primiparous. Most women 79.7 % reported that they had wanted pregnancy, and never experienced abortion (92.3 %). The characteristics of the study sample are presented in Table 1.

**Table 1:** The characteristics of the study sample (*n* = 518)

	Number	%
Age (years)		
Mean (SD)	26.41 (4.71)	
Duration of marriage		
Mean (SD)	5.27 (4.07)	
Employment status		
House wife	461	89.0
Employed	57	11.0
Education		
Primary	167	32.2
Secondary	237	45.8
Higher	114	22.0
Parity		
Primiparity	321	62.0
Multiparity	197	38.0
Unwantedpregnancy		
No	413	79.7
Yes	105	20.3
History of abortion		
Yes	40	7.7
No	478	92.3
Trimester of pregnancy		
1	160	30.9
2	200	38.6
3	158	30.5

### Sexual functioning during pregnancy

The mean score (SD) for desire, excitement and enjoyment was 6.2(+/−2.6), 5.8 (+/− 2.1) and 4.9 (+/− 2.0), respectively. However, scored better for pain during intercourse (mean = 8.8, SD = +/− 1.5) and worry about fetus injury (mean = 13.6, SD = +/− 1.9). The mean score for the total sexual functioning was 39.4 (SD = +/− 5.2). Overall 309 women (59.7 %) scored less than mean. The descriptive findings are shown in Table 2.

**Table 2:** Sexual function scores for the study sample

		Mean (SD)	Possible score range^a^
Sexual satisfaction			
	Desire	6.2 (2.6)	0-16
	Excitement	5.8 (2.1)	0-12
	Enjoyment	4.9 (2.0)	0-12
Sexual worries			
	Dyspareunia	8.8 (1.5)	0-12
	Fetal injury concern	13.6 (1.9)	0-16
Total score			
	Mean (SD)	39.4 (5.2)	0-68
	Range	27-56	
	Women scoring less than mean (n, %)	309 (59.7)	

^a^Higher scores indicate better conditions for all subscales

### Factors affecting couples’ sexual functioning during pregnancy

The results obtained from generalized linear models demonstrated that lower education (B = −3.04, % 95 CI = −3.32 to −2.77), unwanted pregnancy (B = 1.27, 95 % CI = 1.04-1.50), earlier stage of pregnancy (B for first trimester = − 0.50, 95 % CI = −0.72 to −0.28), older age (B = −0.17, 95 % CI = −0.20-0.15), and longer duration of marriage (B = 0.06, % 95 CI = 0.03-0.10) were significant contributing factors to women’s sexual dysfunction. In fact the results obtained from the analysis indicated that women with older age, lower educational level, unwanted pregnancy and being at first trimester were more likely to report disturbed sexual function. The findings are shown in Table 3.

**Table 3:** The results obtained from generalized linear model analysis indicating risk factors for sexual dysfunction (*n* = 518)

	B	Std Error	95 % CI	P
Age (years)	−0.17	0.01	−0.20 to −0.15	<0.001
Employment status				
Employed	Ref.			
House wife	−0.07	0.166	−0.39 to 0.25	0.665
Education				
Higher	Ref.			
Secondary	−1.15	0.12	−1.41 to −0.90	<0.001
Primary	−3.04	0.14	−3.32 to −2.77	<0.001
Parity				
Primiparity	0.26	0.13	−0.53 to 0.008	0.05
Multiparity	Ref.			
Unwanted pregnancy				
Yes	1.27	0.116	1.04 to 1.50	<0.001
No	Ref.			
History of abortion				
Yes	−0.15	0.18	−0.52 to 0.20	0.391
No	Ref.			
Trimester of pregnancy				
1	−0.50	0.11	−0.72 to −0.28	<0.001
2	−0.89	0.10	−1.10 to −0.68	<0.001
3	Ref.			
Duration of marriage	0.06	0.01	0.03 to 0.10	<0.001

## Discussion

We found that unwanted pregnancy could affect sexual function during pregnancy. Perhaps unwanted pregnancy can affect womens’ physical and mental health and thus affects sexual functioning during pregnancy [26, 27]. In addition, it has been suggested that unwanted pregnancy can cause stress. As such emotional and stress-related problems provide high risk of occurring sexual dysfunction [28]. However, it has been suggested that the lack of adequate information about sex in pregnancy and concerns about the possible adverse obstetric outcomes are the most relevant factors responsible for the avoidance of sexual activity during pregnancy [29]. In addition as suggested psychological variables such as relationship satisfaction also play important role in decreased sexual function during pregnancy [16].

The results showed that level of education was a significant contributing factor to the sexual function. It is well established that individuals with high education level are healthier and have less sexual problems [30]. Perhaps one might argue that this could be explained by the fact that since level of education considered as one of the personality-related predisposing factors for help-seeking behaviors [31] therefore it seems that the well-educated women were more likely to seek help for sexual dysfunction during pregnancy.

In the present study we showed that the trimester of pregnancy was contributing factor to the sexual function. In fact the current study pointed out that overall sexual activity was higher in the third trimester compared to the first trimester. This might be attributed to the advise couples received for doing sex at that time in order to improve fetal well-being, and facilitate labor or delivery [4, 32, 33].

The present study confirmed that longer duration of marriage was related to sexual dysfunction. as noted by Eryilmaz and Zincir [34]. This might be due to the fact that duration of marriage perhaps is an indication of sexual relationship between couples even at pre-pregnancy period (before conception). A recent study found that women who had pre-pregnancy sexual dysfunction continued to experience it during pregnancy, and the majority of them had a significant level of sexual dysfunction in the postpartum period [29]. However as suggested a discussion of expected changes in sexuality should be routinely checked by health care team in order to improve couples' perception of possible sexual modifications induced by pregnancy [13].

### Limitations

This study had some limitations. We obtained our sample of women pregnancy from public health services in the Eastern district of Guilan, Iran thereby excluding women who had not used any healthcare facilities. In addition to recall bias that is a central issue in this type of research, the study was cross-sectional and one should not draw causality from the results. Furthermore the study sample was not representative of Iranian pregnant population in term of maternal age, education, parity, and employment. Thus the results could not be generalized and should be interpreted with caution.

## Conclusions

Sexual function in pregnant women might be influenced by several factors including lower education, unwanted pregnancy, earlier stage of pregnancy, older age, and longer duration of marriage. It seems that issues on sexual function during pregnancy should be included as part of prenatal care and reproductive health programs for every woman.
